# Increased levels of circulating IL-10 in persons recovered from hepatitis C virus (HCV) infection compared with persons with active HCV infection

**DOI:** 10.1186/s13104-020-05313-w

**Published:** 2020-10-07

**Authors:** Dorcas Ohui Owusu, Richard Phillips, Michael Owusu, Fred Stephen Sarfo, Margaret Frempong

**Affiliations:** 1 Department of Medical Laboratory Technology, Garden City University College (GCUC), P.O. Box 12775, Kumasi, Ghana; 2 Kumasi Centre for Collaborative Research in Tropical Medicine (KCCR), Kwame Nkrumah University of Science and Technology (KNUST), PMB, KNUST, Kumasi, Ghana; 3 Department of Medical Diagnostics, Kwame Nkrumah University of Science and Technology (KNUST), University Post Office, Kumasi, Ghana; 4 Department of Medicine, Komfo Anokye Teaching Hospital, Kwame Nkrumah University of Science and Technology, University Post Office, Kumasi, Ghana; 5 Department of Molecular Medicine, Kwame Nkrumah University of Science and Technology, Kumasi, Ghana

**Keywords:** Hepatitis C virus, Cytokine, Spontaneous recovery

## Abstract

**Objective:**

Approximately 70% of all hepatitis C (HCV) infections develop chronic disease. Active or exacerbated chronic hepatitis C infection subsequently progress to liver disease. The role of T-cells secretions in achieving viral clearance is still not well understood. Thus, the current study was set to determine the relationship between the T cell cytokine profiles, biochemical parameters and persistent HCV infection or spontaneous recovery.

**Results:**

Twenty-five percent (41/163) of the anti-HCV positive participants had recovered from HCV and had significantly higher concentration of IL-10 compared to those with active HCV infection (P < 0.012). Other circulating cytokines measured; IL-2, IFN gamma, TNF alpha, IL-5 and IL-17 were similar in both groups. Participants with active HCV infection had significantly higher aspartate transaminase (AST) (35 units) and alanine transaminase (46 units) compared to those in the recovered state (P < 0.001). Thus, serum levels of IL10 could be explored in larger prospective cohort study as a predictive marker of recovering from an active HCV infection.

## Introduction

Hepatitis is a leading cause of morbidity and mortality in developing countries. Majority of these liver diseases are known to be of viral origin of which hepatitis C virus (HCV) is the second highest viral causal agent. It is estimated that 0.5% to 3.5% of the world’s population, representing 36–266 million people are infected with hepatitis C [1, 2]. Out of all infected individuals, 54–80% result in chronic forms of hepatitis C and subsequently lead to severe acute liver inflammation and damage [3, 4].

HCV is distributed worldwide, but with uneven geographical spread [5]. In Africa, the overall sero-prevalence is estimated to be between 0 and 66% [5–14]. In Ghana, the prevalence of hepatitis C varies between 0.3 and 23.2% across different locations. [11, 15].

Chronic or active forms of hepatitis C infection is achieved through successful evasion of the body’s immune surveillance system [16–18]. There is limited data on the immunopathology of HCV infection in the Sub-Saharan Africa. The few data available have been from relatively low endemic regions. T-cells are thought to play a role in active HCV infection or spontaneously recovering from it, but the mechanism by which the virus is able to maintain viral perseverance in more than 50% of exposed individuals is still not well understood. Thus, there is the need to determine biomarkers, including serum cytokines which contribute to immunopathogenesis of hepatitis C virus infection and their capability to predict the outcome of HCV infection. The objective of the present study was to determine and analyse the relationship between the T cells cytokine profiles, biochemical parameters and persistence HCV infection or recovery using a single observation design.

## Main text

### Methods

#### Study population/design

**Fig. 1:**
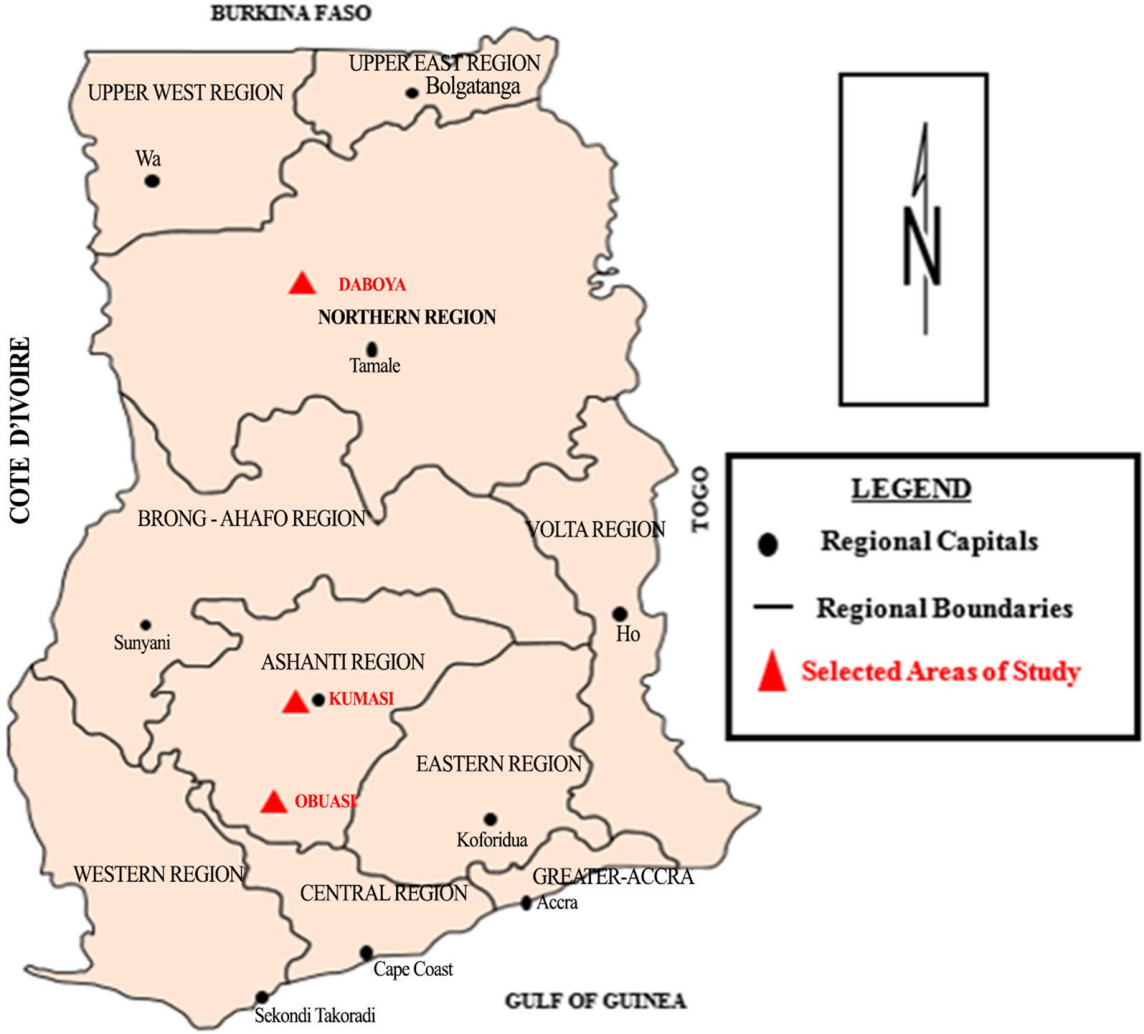
Map of Ghana with selected areas of study. The map was adapted and modified from our previously published manuscript [44]

The study employed cross-sectional observation design. The study population was derived from individuals who had tested for HCV positive based on a rapid serological assay test. These individuals were recruited from three different sites in Ghana; the Transfusion unit of Komfo Anokye Teaching Hospital, Obuasi municipality and Daboya community. We recalled 322 individuals using records obtained from the previous work which was carried out between 2013 and 2014 (unpublished). Komfo Anokye Teaching Hospital (KATH) is the second largest tertiary medical facility in Ghana. The Blood Transfusion Medicine Unit serves KATH and other health facilities in Ashanti Region. Obuasi Municipality is a big cosmopolitan municipal in Ashanti region with varying intercultural background due to ongoing mining activities and an estimated population of 169,000. Daboya community is a district capital with an estimated population of 6,510 in the Northern part of Ghana (Fig. 1).

#### Inclusion and exclusion

Participants who were 18 years and above who voluntarily gave written informed consent were included. Participants should not have initiated HCV treatment at the time of enrolment. Participants who refused to provide consent were excluded from the study.

#### Consent and ethics

Ethics approval was granted by the Kwame Nkrumah University of Science and Technology and the Komfo Anokye Teaching Hospital research ethics review committee (Reference; CHRPE/AP/134/13, CHRPE/AP/443/13 and CHRPE/AP/162/15). Written consent was obtained from all participants above 18 years. Confidentiality of participant information was maintained at each level of the study.

#### Sample collection and processing

At least 5 ml of venous blood samples were collected from all HCV seropositive participants. Blood were collected into EDTA and serum vacutainers, transported to the laboratory and further processed for serum.

#### Anti-HCV screening confirmation

All samples collected were further confirmed for HCV sero-positivity using ORTHO^®^ HCV Version 3.0 ELISA Test System (Ortho Clinicals Incorporated, USA) according to the manufacturer’s protocol. The test determined antibodies to recombinant hepatitis C encoded antigens c22-3, c200 and NS5 regions which is precoated in microwell-plates.

#### HCV quantification

Samples that were confirmed to be anti-HCV positive were further tested for HCV RNA and quantified using the Abbott Real-time RT-PCR (Abbott, IL, USA) automated system according to the manufacturer’s instructions.

#### HBsAg screening and confirmation

All the anti-HCV confirmed positive samples were serologically assessed for hepatitis B virus surface antibody (anti-HBsAg). The test assay was done using GS HBsAg EIA 3.0 (BIO-RAD, Redmond, Washington. USA) and according to the manufacturers protocol.

#### HIV screening

All the anti-HCV confirmed positive samples were further serologically assessed for HIV-1 and HIV-2 antigen and antibodies. The test assay was done using GS HIV Combo Ag/Ab EIA (BIO-RAD Clinical Diagnostic group, Redmond, Washington. USA) and according to the manufacturers protocol. The tests determined antigens and antibodies to HIV 1&2 using microwell-plates precoated with monoclonal antibodies to HIV p24 (mouse) and HIV-1 and HIV-2 antigens.

#### Biochemical analysis

Serum alanine aminotransferase (ALT) and aspartate aminotransferase (AST) levels were measured using ALT/GPT 4 + 1 SL (ELITech Clinical Systems) on Selectra ProS automated chemistry analyser (ELITech company, Japan) per protocol.

#### Analysis of serum cytokines

Samples from participants with known HCV status were analysed for their cytokine levels. The relative expression levels of T cell cytokines (Interleukin 2 (IL-2), Interferon (IFN) gamma, Tumour necrosis factor (TNF) alpha, Th2 (Interleukin 5 (IL-5), Th17 cytokines (Interleukin 17 (IL-17) and Interleukin 10 (IL-10) were determined. Cytokines were quantitated using commercially available ELISA assay (Affymetrix eBioscience ELISA Ready-SET-Go, San Diego–California) according to the manufacturer’s instructions. High protein binding ELISA plates were coated with specific capture monoclonal antibody to determine the cytokines (analytes) in the serum samples.

#### Definitions for hepatitis C infection

For purposes assessing the immunological and biochemical markers in hepatitis C, we classified our study subjects into recovered and active HCV states of infection. Subjects were considered recovered when the serological test was positive but HCV RNA was negative. Subjects were considered as having active HCV infections when the serological test and HCV RNA were both positive. These classifications were based on previous reports [19].

#### Data collection and statistical analysis

Socio-demographic information was collected through face–face interview with participants. The socio-demographic study variables determined were sex, age, use of alcohol and hepatitis B status. All categorical variables were analysed using Chi square test. Continuous variables and their subcategories were also analysed using either parametric or non-parametric methods based on the distribution of the variables. Statistical analyses were performed using IBM SPSS Statistics v.12 (IBM Computer hardware company, New York, USA) and *p* value of ≤ 0.05 was considered statistically significant.

### Results

A total of 163 participants who agreed to participate in the study were confirmed to be HCV sero-positive. Of the 163, 97 (59.5%) were from KATH, 38 (23.3%) were from Obuasi and 28 (17.7%) were from Daboya. Ninety-eight (98; 60.1%) of the study subjects were males and 65 (39.9%) were females. The overall mean age of participants was 36. Table 1 shows the socio-demographic characteristics of participants.

**Table 1:** Socio-demographic characteristics of participants

Total	N = 163
Sex	
Female n (%)	65 (39.9)
Male n (%)	98 (60.1)
Age	
Mean (SD)	35.8 (11.9)
Occupation^a^	
Modern professional occupation n (%)	28 (23)
Farming or agricultural occupation n (%)	13 (10.7)
Routine manual and service occupations n (%)	36 (29.5)
Student n (%)	15 (12.3)
Technical and craft occupations n (%)	24 (19.7)
Unemployed n (%)	6 (4.9)
HBsAg status^b^	
Negative n (%)	146 (90.1)
Positive n (%)	16 (9.9)
HIV status^b^	
Negative n (%)	154 (95.1)
Positive n (%)	8 (4.9)

^a^One participant could not provide information on his occupation

^b^One participant did not agree to be screened for HBsAg and HIV

Of the 163 seropositive participants, 122 (74.84%; 95%CI 67.46–81.30) had active HCV infection and 41 (25.15%; 95%CI 18.69–32.53) were in the recovery state. The mean hepatitis C viral load of participants with active HCV infection was 5.5 log_10_ IU/ml (± 1.0 IU). Table 2 shows a comparison of sex, age, HBsAg status, HIV status, alcohol use and median liver marker enzymes (AST and ALT) between active and recovered HCV infected individuals. The active HCV infected participants had significantly higher AST (35 U/L) and ALT (46 U/L) compared to those in the recovery state (P < 0.001).

**Table 2:** Characteristics and immunological parameters of active and recovered HCV participants

	Recovered HCV (N = 41)	Active HCV (N = 122)	P value
Sex			0.293
Female (%)	13 (31.7)	52 (42.6)	
Male (%)	28 (68.3)	70 (57.4)	
Age (mean, SD)	32 (26,38)	35 (29,43.8)	0.069
HBsAg status			0.546
Negative (%)	35 (87.5)	111 (91)	
Positive (%)	5 (12.5)	11 (9)	
HIV status			0.681
Negative (%)	39 (97.5)	115 (94.3)	
Positive (%)	1 (2.5)	7 (5.7)	
Alcohol use			0.551
No (%)	35 (85.4)	97 (79.5)	
Yes (%)	6 (14.6)	25 (20.5)	
AST (median, IQR) U/L	26 (14.2,31)	35 (25,55)	< 0.001*
ALT (median, IQR) U/L	27 (7.2,41.5)	46 (23,70)	< 0.001*
IL-2 (pg/ml)	132.4 (71.0–207.1)	138.7 (81.8–199.0)	0.718
IL-5 (pg/ml)	44.4 (37.7–76.0)	49.7 (40.7–73.2)	0.549
IL-10 (pg/ml)	67.2 (50.6–113.1)	48.5 (30.3–63.2)	0.012
IL-17A (pg/ml)	18.6 (9.6)	18.2 (8.5)	0.874**
TNF alpha (ng/L)	61.6 (22.3–99.5)	22.7 (0.3–111.9)	0.125
IFN gamma (pg/ml)	59.4 (8.0–194.0)	72.8 (34.1–275.8)	0.573

*AST* Aspartate aminotransferase, *ALT* Alanine aminotransferase, *HBsAg* Hepatitis B surface antigen

Wilcoxon signed-rank test (*), Cytokine levels are presented as median and interquartile range (Q1–Q3) (**). The medians of the groups were compared by the Ranksum test or T-test where appropriate

#### Cytokine responses

In order to determine the variation in the levels of cytokine among study participants, serum samples were analysed for Th1 (IL-2, IFN gamma and TNF alpha), Th2 (IL-5), Th17 (IL-17A) and Treg (IL-10) cytokines. From the analysis, anti-HCV positive participants who had recovered from the infection had significantly higher [67.2 (50.6–113.1) pg/ml] concentration of IL-10 compared to those with active infection (48.5 (30.3–63.2) pg/ml) p = 0.012. All other cytokines were similar for the two groups. The comparison in levels of cytokines between recovered and active HCV infected participants are shown in Table 2.

### Discussion

Acute infection with HCV often begins as asymptomatic and a large proportion of these infected participants are not able to recover completely. They enter into the chronic phase with active infection. The exact mechanisms underlying spontaneous recovery from HCV is unclear. Therefore, we aimed our study at determining some specific serum cytokines that could play a role in spontaneous recovery from HCV infection.

We determined levels of T cell cytokines IL-2, IFN-γ, TNFα, IL-5, IL-10 and IL-17A among active HCV participants and HCV spontaneously recovered participants. Our study results showed significantly increased levels of IL-10 among spontaneously HCV recovered participants compared with active HCV infection. IL-10 is known to account for appropriate balance of the T helper cytokines necessary for the natural elimination of the hepatitis C viruses. This is consistent with observations made by Mangia et al. and Shaker et al., who demonstrated that heterogeneity in the promoter region as well as SNPs of the IL-10 gene influences the determination of spontaneous or treatment induced favourable outcome of HCV infection [20, 21]. IL-10 produced by stimulated regulatory T cells regulates Th1 and Th2 by inhibiting their inflammatory responses [22]. IL10 controls the differentiation and proliferation of B cells, T cells, antigen-presenting cells, mast cells and granulocytes but negatively regulates Natural killer cells. IL-10 promotes development of type 2 cytokines by inhibiting IFN-γ production [23].

Contrary to our study, Osburn et al. reported decreased levels of IL-10 and IL-2 in individuals who had cleared HCV infection among high risk adults as compared to those who had chronic infection [24]. This outcome differs from our study possibly due to the study design, differences in the age groups of the study populations, environmental exposures and host genetic make-up. We did not observe differences in the interferon, IL-17 and IL-2 levels detected in the serum of recovered and actively infected subjects. IL-2 is known to function by promoting the differentiation of immature T cells into effector and regulatory T cells which may positively affect the outcome of infection. [25, 26]. Interferons exhibit antiviral activity, antiproliferative/antitumor and immunomodulatory effects [27]. Despite the apoptotic signalling pathway induced by TNFα, our study found to not play any role in recovery of HCV infected participants. This outcome is similar to the studies by Abdou et al. 2015 and Costantini et al. 2010 [28–30]. Sex and age did not show any significant difference among active HCV infection and spontaneous recovery. This is consistent with observations made by Cho et al. and Kim et al. who could not find any statistically significant role played by sex in spontaneous viral clearance of HCV in a Korean population [31, 32]. Tsui et al. made a similar observation in an adult population [33]. However, Bulteel et al. in their retrospective study identified females within the younger age group showing positive association with spontaneous clearance [34]. The variation could be due to the difference in the geographical location and limited sample size of our study population. In this study there was no statistically significant relationship between spontaneous HCV cleared infection and co-infection with HBV or HIV. But in other studies, co-infection with other viruses had an effect on the natural history of HCV infections leading to variation in detection of serum nucleic acid levels of HCV RNA [35–42]. The study did not established a significant relationship between alcohol consumption and spontaneous HCV clearance or active infection even though alcohol have some immunosuppressive properties [33, 37, 43]. The outcome of the study suggest that serum levels of AST and ALT could be employed as a predictive measure of the status of HCV infection in seropositive subjects.

### Conclusion

In the present study, age, sex, co-infection with hepatitis B or HIV variables were not found to be associated with spontaneous HCV clearance in the selected Ghanaian population. The levels of IL-2, IFN-γ, TNFα, IL-5 and IL-17A in the serum of HCV sero-positive are not associated with active HCV infection or spontaneously recovered infection. The levels of IL-10 was high in spontaneously recovered HCV persons, therefore could be explored as a predictive measure of HCV status in anti-HCV positive persons.

## Limitations

The limitations of this study could be conducting a cross-sectional study and the small sample size used. Longitudinal studies that employs a larger population size could provide useful information that would further strengthen the findings of this study.

## Data Availability

The datasets used and/or analysed during the current study are available from the corresponding author on reasonable request.

## Abbreviations

IL: Interleukin
HCV: Hepatitis C virus
GCUC: Garden City University College
KCCR: Kumasi Centre for Collaborative Research in Tropical Medicine
KNUST: Kwame Nkrumah University of Science and Technology
IFN: Interferon gamma
KATH: Komfo Anokye Teaching Hospital
EDTA: Ethylenediaminetetraacetic acid
CHRPE: Committee for Human Research, Publications and Ethics
ELISA: Enzyme linked immunosorbent assay
RNA: Ribonucleic acid
RT-PCR: Real time polymerase chain reaction
HBsAg: Hepatitis C surface antigens
HIV: Human immunodeficiency virus
